# Implementation of Antibody Rapid Diagnostic Testing versus Real-Time Reverse Transcription-PCR Sample Pooling in the Screening of COVID-19: a Case of Different Testing Strategies in Africa

**DOI:** 10.1128/mSphere.00524-20

**Published:** 2020-07-29

**Authors:** Tinashe K. Nyazika, Rabelani Kaela, Mathias Mugoni, Kudakwashe Musomekwa, Eric Kyei-Baafour, Simbarashe Chiwanda, Prichard T. Mapondera, Tatenda S. Makawa, Elliot M. Sithole, George Mavunganidze, Justen Manasa, Kondwani C. Jambo, Cuthbert Musarurwa

**Affiliations:** a Malawi-Liverpool-Wellcome Trust Clinical Research Programme, University of Malawi College of Medicine, Blantyre, Malawi; b Department of Clinical Sciences, Liverpool School of Tropical Medicine, Liverpool, United Kingdom; c Department of Pathology, College of Medicine, University of Malawi, Blantyre, Malawi; d African Society of Laboratory Medicine, PHIA Lab Corps, Addis Ababa, Ethiopia; e Department of Microbiology, Optimum Health Medical Laboratories, Gaborone, Botswana; f Aids Healthcare Foundation, Bulawayo, Zimbabwe; g Department of Immunology, Noguchi Memorial Institute for Medical Research, University of Ghana, Accra, Ghana; h Department of Epidemiology and Disease Control, Ministry of Health and Child Care, Harare, Zimbabwe; i Department of Paraclinical Veterinary Studies, Faculty of Veterinary Studies, University of Zimbabwe, Harare, Zimbabwe; j Department of Health Sciences, School of Public Health, University of South Wales, Wales, United Kingdom; k Zimbabwe Association of Medical Laboratory and Clinical Scientists, Harare, Zimbabwe; l Department of Medical Microbiology, College of Health Sciences, University of Zimbabwe, Harare, Zimbabwe; m Department of Chemical Pathology, College of Health Sciences, University of Zimbabwe, Harare, Zimbabwe; University of Missouri-Kansas City School of Medicine

**Keywords:** antibody rapid diagnostic test, sample pooling, rRT-PCR, COVID-19, Africa

## Abstract

Coronavirus disease 2019 (COVID-19) has wreaked havoc across the globe; although the number of cases in Africa remains lower than in other regions, it is on a gradual upward trajectory. To date, COVID-19 cases have been reported in 54 out of 55 African countries. However, due to limited severe acute respiratory syndrome coronavirus 2 (SARS-CoV-2) real-time reverse transcription-PCR (rRT-PCR) testing capacity and scarcity of testing reagents, it is probable that the total number of cases could far exceed published statistics. In this viewpoint, using Ghana, Malawi, South Africa, and Zimbabwe as examples of countries that have implemented different testing strategies, we argue that the implementation of sample pooling for rRT-PCR over antibody rapid diagnostic testing could have a greater impact in assessing disease burden. Sample pooling offers huge advantages compared to single test rRT-PCR, as it reduces diagnostic costs, personnel time, burnout, and analytical run times. Africa is already strained in terms of testing resources for COVID-19; hence, cheaper alternative ways need to be implemented to conserve resources, maximize mass testing, and reduce transmission in the wider population.

## Abstract (precis)

Coronavirus disease 2019 (COVID-19) has wreaked havoc across the globe; although the number of cases in Africa remains lower than in other regions, it is on a gradual upward trajectory. To date, COVID-19 cases have been reported in 54 out of 55 African countries. However, due to limited severe acute respiratory syndrome coronavirus 2 (SARS-CoV-2) real-time reverse transcription-PCR (rRT-PCR) testing capacity and scarcity of testing reagents, it is probable that the total number of cases could far exceed published statistics.

## PERSPECTIVE

The severe acute respiratory syndrome coronavirus 2 (SARS-CoV-2) outbreak was first reported in Wuhan, China, in December 2019, and it has spread throughout the world with an unprecedented impact on humanity (1). The disease later termed coronavirus disease 2019 (COVID-19) has now been reported to have affected more than 6.2 million people worldwide, culminating in more than 376,000 deaths by 2 June 2020 (2). In Africa, COVID-19 has been reported in 54 countries, with a cumulative 155,610 confirmed cases and 4,429 deaths (3). With more than 3 billion people under COVID-19-induced lockdown worldwide, accompanied by the attendant negative impact on global economies, governments are racing to flatten the epidemic curve of COVID-19 in both space and time (4, 5). Some of the policies imposed by governments to flatten the curve include travel bans, lockdowns, quarantining, and widespread mass testing using real-time reverse transcription PCR (rRT-PCR) (4, 5).

In order to achieve gains from these implemented measures and to reduce the burden of COVID-19 cases on societies and economies, it is critical to implement diagnostic strategies with high analytical sensitivity and specificity to enable early detection of SARS-CoV-2 cases for isolation, contact tracing, care, and management. International collaborative efforts initiated after the emergence of the SARS-CoV-2 pandemic led to the development of rRT-PCR diagnostic assays to ascertain cases and track the outbreak (5, 6). The rRT-PCR is reported to have a diagnostic sensitivity of 88 to 99% and a diagnostic specificity of 77 to 100% (7). This development thus accelerated the definitive diagnosis of asymptomatic, presymptomatic, and symptomatic cases (6).

Due to limited SARS-CoV-2 rRT-PCR testing capacity and scarcity of testing reagents in many geographical regions, including Africa, it is clear that the total number of COVID-19 cases is probably much higher than published confirmed statistics (8). Using Ghana, Malawi, South Africa, and Zimbabwe as examples of countries that have implemented different testing strategies, we argue that the implementation of sample pooling for rRT-PCR over antibody rapid diagnostic testing (RDT) could have a greater impact on the early detection of active COVID-19 cases and assist in curbing the spread of the disease, whereas antibody-based RDTs may help to ascertain community seroprevalence and estimate potential herd immunity benefits.

### Case studies of COVID-19 diagnostic strategies in Africa.

**(i) A case study of Ghana.** Ghana is one of the few countries that implemented sample pooling strategy for COVID-19 rRT-PCR testing and confirmation (9, 10). However, at the onset of the pandemic, the country implemented single sample rRT-PCR testing before scaling it upwards through sample pooling (10, 11). As of 31 May 2020, a total of 219,825 tests had been conducted by the seven government-affiliated laboratories spread across Ghana (12). Through sample pooling, approximately ∼0.71% of the total population have been tested, giving rise to 8,297 confirmed COVID-19 cases (positivity rate of 3.77%) (12). Ghana is the second highest ranked African country in terms of testing capacity. The COVID-19 testing strategy implemented pooled at most 10 different patient samples for amplification prior to testing. This strategy has tremendously improved Ghana’s COVID-19 testing capacity and definitive diagnosis.

**(ii) A case study of Malawi.** As of 1 June 2020, Malawi had conducted 5,505 rRT-PCR tests, yielding 358 COVID-19 cases (13). Malawi has the highest SARS-CoV-2 positivity rate (6.50%) in the region, despite its low testing rate of only ∼0.03% of the population tested so far (13). The country recently increased its COVID-19 testing sites to 14 in order to expand geographic coverage. Owing to the low testing capacity, Malawi needs to be more restrictive in choosing who to test in order to maximize benefits from its limited resources.

**(iii) A case study of South Africa.** South Africa has conducted 742,742 rRT-PCR tests, yielding 35,812 COVID-19 cases and a positivity rate of 4.82% (14). It is the leading African country in terms of testing capacity, with 1.25% of its population having been tested for COVID-19 by 1 June 2020. South Africa is currently conducting individual sample testing using several in-house and commercial PCR assays to test for the presence of SARS-CoV-2 RNA (15). However, most diagnostic laboratories are overwhelmed, as the country is conducting targeted community symptom screening and testing for both symptomatic and asymptomatic individuals in some provinces (15, 16). Novel screening and testing strategies need to be adopted in order to manage and improve the capacity and sample turnaround testing time in diagnostic laboratories.

**(iv) A case study of Zimbabwe.** Recently, the Ministry of Health and Child Care (MoHCC) of Zimbabwe introduced antibody RDT as a screening test strategy for SARS-CoV-2 to complement the rRT-PCR assays being offered in government reference laboratories, state universities, and private laboratories. Despite this development, the country still has limited capacity to conduct mass testing (17). As of 1 June 2020, a total of 46,021 COVID-19 tests (28,112 RDTs and 18,709 RT-PCR) had been conducted, with 203 confirmed cases (18). The current testing coverage translates to approximately ∼0.13% of the population tested by rRT-PCR/Xpert Xpress for SARS-CoV-2 with a 1.09% positivity rate. Furthermore, many of the RDTs in current use have not been validated or verified for use within this population. Thus, the current rRT-PCR undertesting raises the concern that many infected individuals are probably going undiagnosed.

### Implementation of different testing strategies for COVID-19.

**(i) Sample pooling for rRT-PCR.** Sample pooling was first suggested by Robert Dorfman in 1943 as a way of optimizing syphilis testing, and since then, it has been applied in surveillance and screening for infections such as human immunodeficiency virus (HIV), hepatitis B virus (HBV), hepatitis C virus (HCV), malaria, and influenza virus (19–25). Sample pooling has popularly been employed in serological work but also in molecular diagnostics and research. It helps to lower experimental costs and personnel time and reduces analytical run times while maximizing the level of surveillance and accuracy of the diagnostic test (19, 21, 24, 26). In public health emergencies, such as the COVID-19 pandemic, rapidly growing workloads can outstrip laboratory testing capacity, resulting in reagent shortages and personnel burnout (16, 27). Thus, in large populations with a low disease prevalence (<10%), sample pooling provides a cost-effective strategy for screening asymptomatic and presymptomatic individuals (25, 27–29). Two different sample pooling strategies can be used, namely, single-step pools, where prevalence and confidence intervals are calculated based on pool results (30), or multiple-step pools, where individual samples from a positive pool are retested to identify the number of positive individuals per pool (31, 32). The latter is more useful for COVID-19, as the individuals need to be identified for isolation, contact tracing, care, and treatment.

The use of a single test to screen groups of more than two people, though labor intensive, provides a comprehensive, rapid, and localized mass testing strategy required to identify cases acutely and minimize spread of disease. However, the anticipated cost reduction becomes meaningful only if statistical equivalence between the pooled and nonpooled experimental setups is achieved and maintained (33). *In vitro* and *in silico* studies of pooling nasopharyngeal samples for rRT-PCR assays, have reported the optimal pool size (*p*), to be five samples per pool (28, 29). On account of variances in different PCR assay limits of detection (LoD), it is paramount to validate the use of pooled samples and rule out false-negative findings as a result of sample dilution. Of note, *p* can be scaled up to *p* = 32 (29, 34–36), where the concentration of RNA by elution/lyophilization becomes necessary to curb the risk of false-negative results due to sample dilution (37). Fundamental principles for successful application of sample pooling are subject to the assay’s sensitivity, specificity, and limit of detection and the prevalence of disease in the population.

COVID-19 is estimated to have a high effective reproductive number *R*_0_ (ranging from 1.4 to 6.49, with a median value of 2.79) (38). Employing sample pooling for contact tracing ensures swift case identification, with less resources used to arrest community transmissions (38–40). Meanwhile, for frontline health care workers (HCW) interfacing with COVID-19 patients, sample pooling can be modeled to cluster high-risk individuals in order to concentrate the positivity rate to a few pools, thereby further conserving resources.

Sample pooling is also associated with limitations such as reduced test sensitivity in settings with very low infection prevalence (21, 41). Thus, a diagnostic laboratory has to optimize the pool size based on the prevalence of the infection within the society. Additionally, it is also critical for laboratories to understand that a negative pool result would not distinguish between a true negative and an indeterminate or inconclusive result due to poor specimen collection or handling (24).

**(ii) Antibody RDT testing for COVID-19.** Any acceptable screening or first-line test should ideally have a high clinical diagnostic sensitivity, whereas the consecutive confirmatory test needs to have a high specificity (5, 7, 8, 42, 43). Despite this, some low-medium income countries (LMICs) in Africa have implemented unverified RDTs for COVID-19 into their testing and surveillance protocols. Whether these RDTs are yielding intended results remains to be verified. However, cheaper but well-validated serological assays are essential to complement the fairly expensive rRT-PCR diagnostic assays, to accurately assess the COVID-19 disease burden within communities and map global exposure (6). Samples can also be pooled as is applied when screening blood products for infections such as HBV and HCV to achieve maximum testing benefit (44).

Testing of specific antibodies against SARS‐CoV‐2 in patient blood is a good choice for rapid and simple diagnosis of COVID‐19 (45). However, early studies have reported that the majority of COVID-19 patients seroconvert between days 7 and 11 after exposure to SARS-CoV-2, thus rendering antibody testing questionable in the setting of an acute illness (43, 46, 47). Moreover, RDT kits for SARS-CoV-2 antibodies have an analytical sensitivity of 69 to 88% for IgM and 90 to 99% for IgG (7, 8, 42, 45). Therefore, RDTs pose the following questions: When to test? Whom to test? What to test? How often to test? What to do with test results? (43). Although validated RDTs can be used in seroprevalence studies, data generated from the use of RDTs may not be reliable unless conducted systematically and periodically due to the wide variation in duration of time to seroconversion among individuals (16).

### Scenarios where rRT-PCR sample pooling or antibody RDT may be employed.

**(i) Establishing the status of an asymptomatic individual.** COVID-19 follows an asymptomatic disease course before symptoms appear, thus causing increased spread of the disease (48, 49). Since antibodies can be detected only 6 to 29 days after symptom onset, antibody RDTs may result in false-negative COVID-19 status, which will in turn delay management and contact tracing, thus allowing propagation of infections within communities. On the other hand, various studies using rRT-PCR have demonstrated asymptomatic carriage in obstetric patients (13.7%), Diamond Princess ship passengers (17.9%), and skilled nursing facility residents (35.6%) (48–50). It should, however, be stressed that in the highlighted studies, the asymptomatic carriers contributed ∼50% of the COVID-19 cases.

**(ii) Testing health care workers and other employees providing essential services.** Health care workers and essential service employees showing no symptoms but continuously interacting with the general population have been shown to be capable of transmitting SARS-CoV-2, as demonstrated in the skilled nursing facility residents in New York (48). Mass testing for asymptomatic HCWs and essential service employees is therefore critical in order to mitigate workforce depletion by unnecessary quarantine, reduce spread of atypical, mild, or asymptomatic cases, and protect the health care and essential service workforce (51). Thus, it is crucial to set a reasonable testing schedule and frequency using pooled sampling rRT-PCR after assessing their risk profile to allow early detection and intervention in asymptomatic and presymptomatic individuals. This can be done by splitting employees working in the same department into groups and staggering testing of these groups to help identify any potential circulation (thus need of contact tracing) of the disease among staff while minimizing once off use of resources.

Asymptomatic and presymptomatic HCWs and essential service workers are an underappreciated potential source of infection and worthy of testing to reduce in-hospital transmission and community spread (51, 52). Workers returning to work may be tested by validated RDTs as a means of tracing possible missed asymptomatic/presymptomatic and symptomatic cases. Furthermore, routine temperature checks must be conducted daily; collectively, this will aid in reducing community spread.

**(iii) Contact tracing.** Elucidation of the chain of infection and identification of the source of COVID-19 infections are crucial for effective disease containment (52, 53). Although the rRT-PCR option offers a diagnostic solution and is important for establishing infection status in contacts of an index case, this approach might not be diagnostically useful in patients who have recovered and are no longer shedding the virus (53). The duration of viral shedding for COVID-19 remains uncertain (54), but data from SARS-CoV indicate that 21 days after symptom onset, 53% of cases achieved viral clearance in nasopharyngeal aspirate samples (55). Thus, serological tests are more useful in identifying convalescent cases, ascertaining seroprevalence, and an accurate denominator for the case fatality rate (53).

**(iv) Establishing status in symptomatic patients.** It is critical to establish the status of any person exhibiting COVID-19-related symptoms as soon as possible to enable appropriate management. As previously emphasized, RDTs have a limitation as far as detection at early onset is concerned and thus may not be very useful.

**(v) Establishing past exposure and immunity to COVID-19.** It is critical to identify individuals with past exposure or those that have recovered from SARS-CoV-2 by testing for IgM/IgG antibodies. However, whether the immune response following exposure to SARS-CoV-2 is long-lasting and protective against reinfection remains an issue of debate (5, 47, 56). Furthermore, diagnostic tests that detect antibodies to SARS-CoV-2, including rapid immunodiagnostic tests, need extensive validation to determine their clinical utility (57).

### Conclusions.

The use of sample pooling for rRT-PCR testing particularly in Africa, to screen for active COVID-19 cases has a great advantage over single test rRT-PCR, as it helps lower diagnostic costs, personnel time, and burnout and also reduces analytical run times (27, 29, 37, 48, 58). Africa is already strained in terms of testing resources for COVID-19; hence, cheaper alternatives need to be implemented to conserve resources, maximize mass testing, and reduce transmission in the wider population. Currently, WHO does not recommend the use of antibody- and antigen-detecting rapid diagnostic tests for patient care but encourages their extensive validation to establish their usefulness in disease surveillance and epidemiologic research (57, 59). Health care workers and other essential service workers, particularly those working in cities and towns with confirmed cases, are a key reservoir for the transmission of COVID-19 due to their interface with patients and the wider population, respectively. Thus, it is crucial to set a reasonable testing schedule and frequency using pooling of samples for rRT-PCR after assessing their risk profile to allow early detection and intervention in asymptomatic and presymptomatic individuals. Blanket testing of asymptomatic front-line staff is a futile exercise that will not add value to this fight.

### Key terms and definitions.

Asymptomatic, laboratory-confirmed COVID-19 case who does not display symptoms; presymptomatic, an individual who has been exposed to the virus (becoming infected) but has not developed symptoms yet; seroconversion, the transition from a seronegative condition—where no antibodies are detectable in the serum or are present at titers below the limit of detection— to a seropositive condition, in which antibodies are detectable in serum samples; symptomatic, an individual who has developed signs and symptoms compatible with COVID-19; sensitivity, the ability of a diagnostic test to correctly identify all patients with the disease; specificity, the ability of a diagnostic test to correctly identify all patients who do not have a disease.
